# Three Adult Cases of *Listeria monocytogenes* Meningitis in Vietnam

**DOI:** 10.1371/journal.pmed.1000306

**Published:** 2010-07-27

**Authors:** Tran Thi Hong Chau, James I. Campbell, Constance Schultsz, Nguyen Van Vinh Chau, To Song Diep, Stephen Baker, Nguyen Tran Chinh, Jeremy J. Farrar, H. Rogier van Doorn

**Affiliations:** 1 Hospital for Tropical Diseases, Ho Chi Minh City, Viet Nam; 2 Oxford University Clinical Research Unit, Wellcome Trust Asia Research Programme, Hospital for Tropical Diseases, Ho Chi Minh City, Viet Nam; 3 Centre for Tropical Medicine, Nuffield Department of Clinical Medicine, University of Oxford, Centre for Clinical Vaccinology and Tropical Medicine, Oxford, United Kingdom; 4 Centre of Poverty-related Communicable Diseases, Academic Medical Centre, Amsterdam, The Netherlands; Chinese University of Hong Kong, China

## Abstract

Rogier van Doorn and colleagues from Ho Chi Minh city, Vietnam present a Learning Forum involving three unusual cases of patients with listerial meningitis.

## Description of Cases

**Patient 1:** A 77-y-old male from Ho Chi Minh City (HCMC), Viet Nam, with a history of (compensated) cirrhosis and noninsulin-dependent diabetes, presented to a district hospital with a 3-d history of fever and headaches, followed by unconsciousness. A lumbar puncture (LP) was performed (Table 1), and he was diagnosed with clinically suspected bacterial meningitis and treated with 4 g/d ceftriaxone and 500 mg amikacin. Bacterial culture was negative. His condition deteriorated and he was transferred to the Hospital for Tropical Diseases (HTD) HCMC on day 8 of illness. On admission, he had a Glasgow Coma Scale (GCS) of 8, neck stiffness, atrial fibrillation, and ascites. Cerebrospinal fluid (CSF), taken after normal fundoscopy, and blood laboratory values are shown in Table 1. The Gram and Ziehl-Neelsen (ZN) stains were negative. His chest X-ray showed bilateral infiltrations, suggestive of pneumonia.

**Table 1:** Demographic, history, physical examination, and laboratory data from three patients with listerial meningitis on admission to our hospital.

Data	Case			Normal Range
	1	2	3	
**Sex**	Male	Male	Female	—
**Age (y)**	77	30	34	—
**Clinical**				
Day of illness	8	9	3	—
Neck stiffness	+	+	+	—
Headache	+	+	+	—
Fever	+	+	+	—
Vomiting	NA	+	+	—
Axillary temperature (°C)	NA	37.5	37	—
Pulse (/min)	NA	84	80	—
Blood pressure (mm Hg)	NA	160/80	90/60	—
Respiratory rate (/min)	NA	32	20	—
GCS	8	11	9	—
**CSF referring hospital**				
Day of illness	5	5	—	—
White cells (10^6^ cells/l)	3,100	960	—	<10
Neutrophils	90%	75%	—	—
Lymphocytes	10%	25%	—	—
Red cells (10^6^ cells/l)	20,000	2,700	—	<10
Glucose CSF (mmol/l)	3.6	0.3	—	2.2–3.8
Glucose blood (mmol/l)	ND	6.5	—	3.9–6.4
Glucose ratio blood/CSF	ND	0.05	—	>50%
Protein (g/l)	4	2.5	—	<0.45
**CSF**				
Colour	Orange	Yellow	Yellow	—
Opening pressure (cm H_2_O)	4,5	17	>40	7–18
White cells (10^6^ cells/l)	1,145	600	46	<10
Neutrophils	70%	49%	69%	—
Lymphocytes	30%	51%	31%	—
Red cells (10^6^ cells/l)	15,750	120	0	<10
Glucose CSF (mmol/l)	6.43	0.8	0.6	2.2–3.8
Glucose blood (mmol/l)	8.55	5.3	4.7	3.9–6.4
Glucose ratio CSF/blood	0.75	0.15	0.13	>50%
Lactate (mmol/l)	4.1	8.9	9.5	0.6–2.4
Protein (g/l)	4.1	1.98	1.87	<0.45
Gram stain	Neg	Neg	Neg	—
Bacterial culture	Pos	Pos	Pos	—
ZN stain	Neg	Neg	Neg	—
India Ink stain	Neg	Neg	Neg	—
**Blood**				
Hematocrit	28.4%	36.2%	33.0%	35–45
White cells (10^9^ cells/l)	12.1	20.5	19.2	5–10
Neutrophils	92.4%	87.7%	93.0%	44–66
Lymphocytes	3.4%	4.0%	3.6%	23–43
Platelets (10^9^ cells/l)	169	352	449	150–400
ALT (U/l)	50	48	24	<40
AST (U/l)	34	165	12	<40
Albumin g/l	29.7	NA	NA	35–50
Bilirubin (µmol/l)	15.7	20	14	0–17
HIV test	Neg	Neg	Neg	—
Bacterial culture	Neg	Neg	Neg	—

NA, not available; ND, not determined; ALT, alanine aminotransferases; AST, aspartate aminotransferase.

**Patient 2:** A 30-y-old male engineer from HCMC was admitted to a district hospital with fever, headache, and vomiting on the fourth day of illness. A LP was performed (Table 1), and he was diagnosed with bacterial meningitis of unknown cause and treated with 4 g/d of ceftriaxone for 4 d and 400 mg of intravenous ciprofloxacin for 1 d. CT scan showed diffuse cerebral oedema and hypodensities in the cerebellum. Blood and CSF culture was negative. He became unconscious and was transferred to HTD on the ninth day of illness. On admission he had neck stiffness and a GCS of 11. A LP was performed after intravenous dexamethasone and mannitol (to reduce oedema) were administered: the CSF was yellow with an opening pressure of 17 cm H_2_O. Gram and ZN stains were negative. 600 white cells/µl, a low glucose, and high lactate were noted for the CSF (Table 1). His chest X-ray was normal.

**Patient 3:** A 34-y-old housewife from HCMC presented with five episodes of fever, headache, and vomiting, each lasting between 5 to 7 d over a 3-mo period. 3 d prior to admission, she had a similar episode complicated by confusion and she presented to HTD. On admission she had a stiff neck with a GCS of 9. A CT scan on admission showed mild dilatation of the lateral ventricles and the temporal horns, but no features suggestive of obstructive hydrocephalus. A LP was performed: the CSF was yellow, the opening pressure was >40 cm H_2_O, with 46 white cells (69% neutrophils)/µl with a low glucose and high lactate (Table 1). Gram and ZN stains were negative. Her chest X-ray was normal.

## At This Stage, What Was Our Differential Diagnosis?

HTD is the main referral hospital for meningitis and encephalitis for the whole of southern Vietnam (>40 million people). Differential diagnosis of meningitis-like syndromes in HIV-negative adults like these three patients is highly dependent on geographical, seasonal, and demographic data.

The three patients presented were seen within a period of 1 y. Of the ∼500 patients with a syndrome of fever, headache or neck-stiffness, and GCS alterations who are admitted to HTD each year, approximately 50% are diagnosed with viral encephalitis on the basis of blood and CSF values, especially a relatively low CSF white cell count (<1,000/µl), in combination with normal CSF glucose (>50% of blood level), and normal CSF protein and CSF lactate. Herpes simplex virus, Japanese encephalitis virus, and Dengue virus are the most frequently diagnosed aetiologies.

The other 50% of patients admitted each year have a purulent meningitis. In a previous study, of these patients, approximately 56% had acute bacterial meningitis with *Streptococcus suis* as the predominant pathogen [1]: 33% have tuberculous meningitis, 5% have cryptococcal meningitis, and 5% eosinophilic meningitis (usually caused by *Angiostrongylus cantonensis* or *Gnathostoma spinigerum*) [2].

## Which Routine Test Results Are Now Helpful?

In bacterial meningitis there is characteristically a peripheral leucocytosis and marked pleiocytosis in the CSF with neutrophilic granulocytes as the predominant white cell. In tuberculous and cryptococcal meningitis, lymphocytes predominate in the CSF, and in eosinophilic meningitis, eosinophilic granulocytes. However, early in the course of disease, neutrophils may predominate in CSF with all these aetiologies [3],[4].

Microscopy and culture of CSF can distinguish the different causes of purulent meningitis: normal pyogenic bacteria are visualised with Gram stain, mycobacteria with ZN stain, and cryptococci with India ink stain of microscopy slides. Sensitivity of microscopy and culture for pyogenic bacteria in a referral setting in Vietnam is limited because of widespread preadmission use of antibiotics either self-prescribed over the counter or administered in the primary hospital as in these three patients above. A diagnosis of eosinophilic meningitis is suspected by eosinophilia in CSF and/or peripheral blood and is supported by serology.

Routine haematology and biochemistry was done on peripheral blood and a LP was performed. The results are shown in Table 1. Gram, ZN, and India Ink stains of CSF were negative. Bacterial blood and CSF cultures were performed.

Sensitivity of microscopic diagnosis of tuberculous meningitis is low and culture can take several weeks for a confirmatory result. Moreover, patients with tuberculous meningitis do not always typically present with a purulent meningitis with lymphocytic predominance, but early in the course of disease may present with <1,000 cells/µl in the CSF with a neutrophil predominant profile. As rapid diagnosis and treatment is critical but difficult in tuberculous meningitis, we use a validated diagnostic algorithm for new patients, on the basis of age, duration of illness, blood white cell count, CSF white cell count, and percentage of neutrophiles to diagnose tuberculous meningitis [2].

## Why Did We Think That Bacterial Meningitis in Patients 1 and 2 and Tuberculous Meningitis in Patient 3 Were the Most Likely?

In our setting, rapid diagnosis and treatment of bacterial, tuberculous and cryptococcal meningitis, and of herpes encephalitis are most important, as these are treatable diseases with a rapid and unfavourable course when left untreated or if treatment is delayed.

CSF results from the referring hospital from patients 1 and 2 both showed contamination with red cells, suggestive of a traumatic LP. White cells were relatively high, with neutrophil predominance and high protein in patient 1. In patient 2, the glucose CSF/plasma ratio was very low; this was not the case in patient 1. Microscopy and cultures were negative, but we have no data on antibiotic treatment prior to transfer to our hospital. A diagnosis of bacterial meningitis was made in both patients and seemed justified considering the clinical and laboratory findings. CSF findings at HTD supported this diagnosis. On the basis of the high CSF lactate and protein and the relatively high number of white cells in CSF and peripheral blood, even after several days of antibiotic treatment, a clinical diagnosis of bacterial meningitis with unknown aetiology was made, and treatment with intravenous ceftriaxone was continued. Ceftriaxone remains the drug of choice in a setting of treated meningitis with negative cultures and no apparent recovery, as all the typical organisms associated with uncomplicated community-acquired meningitis in Vietnam are ceftriaxone susceptible. In patients with underlying illness or immunocompromised patients, an aminoglycoside or carbapenem is added to cover *Enterobacteriaceae*. Oxacillin or vancomycin is added to ceftriaxone when *Staphylococcus aureus* is suspected, e.g., post-traumatic or in patients with CSF shunts. Ampicillin is not part of empiric or second line treatment in Vietnam.

A physician from Pham Ngoc Thach Hospital for Tuberculosis and Lung Disease HCMC was consulted for patient 3, and after review, a probable diagnosis of tuberculous meningitis was made on the basis of the relatively low number of CSF white cells despite the apparently short history, and on the high CSF protein and low glucose. Tuberculous meningitis is also extremely common in this setting with over 800 patients seen each year between these two hospitals.

**Patient 1:** Ceftriaxone was continued, and amikacin was changed to 240 mg of gentamicin. After 3 d, culture of the CSF was suggestive of *Listeria spp*.

**Patient 2:** A clinical diagnosis of bacterial meningitis was made, ceftriaxone was continued, and 0.4 mg/kg dexamethasone daily was added. He deteriorated and required mechanical ventilation, then developed multi-organ dysfunction, and died 6 d later. On the day of his death the CSF culture became positive with Gram-positive rods, suggestive of *Listeria spp*.

**Patient 3:** A diagnosis of probable tuberculous meningitis was made, and the patient was transferred to Pham Ngoc Thach Hospital for Tuberculosis and Lung Disease. She was treated for tuberculous meningitis according to standard guidelines with streptomycin, rifampicin, pyrazinamid, isoniazid, and dexamethasone. After 48 h the bacterial CSF culture taken at HTD was suggestive of *Listeria spp*.

## What Is the Meaning of This Test Result?

All three CSF cultures were subsequently confirmed to contain *Listeria monocytogenes* (see below), a bacterium known as a human pathogen since the early 20th century [5]; the foodborne transmission of *L. monocytogenes* was first described in 1983 following a foodborne outbreak in Canada, caused by contaminated coleslaw [6]. The latest major listeriosis outbreak, and the largest recorded to date with 20 deaths, was also reported from Canada and was due to contamination in a meat processing plant [7].

*Listeria spp* have a ubiquitous worldwide distribution, but only *L. monocytogenes* is pathogenic for humans. Unpasteurized dairy products, such as raw milk and soft cheeses, and preprocessed foods are reported to be especially associated with listerial infection; this is caused by Listeria's ability to survive and replicate in cool environments and to produce biofilms, thereby outgrowing other bacteria during storage of food and allowing for cross-contamination of previously uncontaminated foods stored in the same space. Their ubiquity and resistance against low temperatures causes regular exposure of humans in developed countries where food is regularly preprocessed, cooled, and packaged before consumption. Despite their abundance, relatively few cases and incidences of 0.1%–11.3% of all confirmed bacterial meningitis cases from these countries are reported [8]–[15].

There are few reports of listerial infection from the developing world, mostly from Africa and Middle East countries, where raw dairy products are part of a regular diet, and only very few reports from Southeast Asia [16],[17], where both dairy and preprocessed food are not part of the traditional cuisine. These are the first three confirmed cases of bacterial meningitis caused by *Listeria spp* in Vietnam.

A high inoculum of ∼10^9^ is required to produce disease in healthy mammals [18]. Immunosuppression or alkalinization of the stomach may facilitate infection [19]. The incubation time for invasive disease is relatively long, and is reported to range from 11 to 70 d [11].

*L. monocytogenes* causes two forms of invasive disease: a bacteremia or sepsis-like syndrome and a central nervous system infection that can present either as an acute meningitis, a subacute meningoencephalitis, or a brain abscess. The disease has been predominantly reported in pregnant women, and at the extremes of life: in neonates through vertical transmission and in elderly or immunocompromised patients; with a mortality of 20%–30% despite appropriate antimicrobial treatment [20]–[22]. Diagnosis and treatment of listerial CNS infections is hampered by difficulties in visualizing the bacteria in Gram-stains of clinical specimens, their slow growth, and the lack of antilisterial activity in most empiric antimicrobial meningitis regimens, which are usually based on a third generation cephalosporin, which is not effective against *L. monocytogenes*.

In our previous large studies on bacterial meningitis [1], no *Listeria* was isolated, and with the exception of sporadic case reports [17],[23],[24] and a single case record report study from Singapore [16], no systematic data are available on the incidence of listeriosis in Southeast Asia.

At the microbiology laboratory of HTD, *L. monocytogenes* was identified in all patients using colony Gram-stains, positive catalase reaction, β-hemolysis on blood agar, synergistic hemolysis with *Staphylococcus aureus* in the CAMP-test, agglutination, and growth at 4°C. In addition, the specific movement pattern, referred to as “tumbling motility,” was microscopically observed in a day-old room temperature culture (Video S1).

## What Is the Standard Treatment and What Were the Next Steps in Managing This Condition?

Third generation cephalosporins including ceftriaxone are ineffective against *Listeria spp*. The recommended treatment for *L. monocytogenes* meningitis is high dose intravenous ampicillin or penicillin G. Alternatively, a combination of trimethoprim and sulfamethoxazole can be given [3]. Literature on treatment of resistant bacteria is sparse. Fluoroquinolones, aminoglycosides, and rifampicin are reported to have some antimicrobial effect on *Listeria spp*.

No previous cases of listeriosis of the CNS have been recorded in HCMC and no cases were diagnosed in our previous large studies on bacterial and tuberculous meningitis, and hence empirical treatment of meningitis in Vietnam does not contain drugs with antibacterial activity against *Listeria spp*. Listeria infection in the developed world is most often diagnosed at the extremes of life (i.e., neonates and elderly) and among immunocompromised patients, with consequences for empirical treatment in these groups. From our patients, only patient 1 fitted this profile. It is striking that patients 2 and 3 were both healthy adults, with no predisposing conditions rendering them more susceptible to *Listeria* infection. Therefore, on the basis of these three cases, it is difficult to assess what the consequences for empiric treatment should be. At present, ampicillin is given infrequently in addition to ceftriaxone in case of slow recovery or worsening.

**Patient 1:** Ceftriaxone was replaced with 12 g of ampicillin. On the same day the patient developed acute respiratory failure and gastrointestinal bleeding and was mechanically ventilated. The chest X-ray showed progression of the bilateral infiltrates and culture of a broncheoalveolar specimen grew *S. aureus* that was treated with 1,500 mg of vancomycin. Despite treatment, his condition worsened. With a GCS of 4 he developed haemorrhoidal and upper gastrointestinal bleeding, and despite further intensive care he died on the 12th day of illness.

**Patient 3:** The patient was transferred back to HTD with a GCS of 15, and persistent neck stiffness. Initially the bacteria appeared ampicillin- and trimethoprim/sulfamethoxazole-resistant in the laboratory, and treatment with 400 mg of intravenous ciprofloxacin was started, with 0.4 mg/kg dexamethasone. When the definitive susceptibility testing results showed susceptibility to ampicillin, the ciprofloxacin was replaced with 12 g of ampicillin.

The subacute clinical course and CSF results of *Listeria* meningitis can resemble tuberculous meningitis, an infection that is highly prevalent in Vietnam, both in HIV-positive and –negative patients. This diagnostic confusion led to patient 3 being treated for TB meningitis. Treatment with rifampicin and streptomycin included in the standard tuberculous meningitis regimen may be partially effective against *Listeria*.

## What Was the Outcome of This Case?

**Patient 3:** On day 11 of ampicillin treatment, the patient developed fever, axillary and cervical lymph nodes, a facial butterfly-shaped rash, and a rash on her neck and upper chest. An allergic reaction against ampicillin was suspected and treatment was stopped. The rash subsided and no relapse of her meningitic syndrome was recorded on follow-up visits. After recovery she reported frequent consumption of raw milk and soft cheeses, prior to her illness. We have no explanation for her history of episodes of fever and headaches, and after her successful recovery this was not further investigated. She has not experienced a similar episode since.

Listeriosis is typically associated with raw dairy products and food processing involving cooling and storage for several days (allowing for specific growth and enrichment of *Listeria*). In traditional Vietnamese cuisine, raw dairy products are rarely used, processed foods are not widely available, most food is cooked just prior to consumption, and most people do not own a refrigerator. Recently, however, supermarkets and imported dairy products are gaining in popularity and increasing wealth allows more people to have a refrigerator at home. As disease patterns change in Asia, gastric and duodenal ulcer diseases are increasing and more people are taking H2 antagonists and proton pump inhibitors reducing the potential protective acidity of the stomach. In addition, life expectancy changes, increasing incidence of diabetes, and the high prevalence of chronic liver disease and cirrhosis due to hepatitis B virus infection provide an additional population with potentially increased susceptibility to *Listeria* infection, and we anticipate identifying *Listeria* with increasing frequency. These developments should have consequences for diagnostic algorithms and empirical treatment regimes in Vietnam and other countries in Asia as dietary habits and comorbidities change across the region [25],[26].

The patients in this manuscript have given written informed consent (as outlined in the PLoS consent form) to publication of their case details.

**Key Learning Points**

- Listeriosis is a foodborne infection caused by *Listeria monocytogenes* that presents with a sepsis-like syndrome or as an acute-to-subacute central nervous system infection, mostly meningitis, with a 20%–30% mortality despite adequate treatment.
- Listeriosis in the developed world has most commonly occurred at the extremes of life (in neonates by vertical transmission or in the elderly), during severe immune suppression, and especially during pregnancy. This pattern may be different in developing countries: two of the three cases presented here did not match this profile.
- *Listeria spp* are ubiquitous in the environment worldwide and are one of the few bacteria that can grow at 4°C and are, therefore, selected during food processing and storage; raw milk and soft cheeses are the most frequently insinuated products in outbreaks, but produce, meats, and any processed foods can also be a source.
- Changing dietary habits and comorbidities in rapidly developing countries in Asia may lead to changes in the commonly observed pathogens requiring changes in empiric treatment protocols.

## Supporting Information

Video S1 **Hanging drop slide of an overnight brain heart infusion broth inoculated with** ***Listeria monocytogenes*** **from case 3.** In the video the bacteria rotate slowly in and out of focus around a central axis; a characteristic and specific movement pattern referred to as “tumbling motility” (magnification ×400).
(5.27 MB MOV)

## Abbreviations

CSF: cerebrospinal fluid
GCS: Glasgow Coma Scale
HCMC: Ho Chi Minh City
HTD: Hospital for Tropical Diseases
LP: lumbar puncture
ZN: Ziehl-Neelsen
